# Gender differences in access to community-based care: a longitudinal analysis of widowhood and living arrangements

**DOI:** 10.1007/s10433-022-00717-y

**Published:** 2022-07-27

**Authors:** Stefania Ilinca, Ricardo Rodrigues, Stefan Fors, Eszter Zólyomi, Janet Jull, Johan Rehnberg, Afshin Vafaei, Susan Phillips

**Affiliations:** 1 European Centre for Social Welfare Policy and Research, Vienna, Austria; 2 ISEG Lisbon School of Economics and Management, University of Lisbon, Portugal Lisbon,; 3 Aging Research Center, Karolinska Institutet, Stockholm, Sweden; 4 School of Rehabilitation, Queen’s University, Kingston, Canada; 5 Department of Family Medicine, Queen′s University, Kingston, Canada

**Keywords:** Long-term care, Bereavement, Informal caregiving, Europe, REWB models

## Abstract

Persistent inequalities in access to community-based support limit opportunities for independent living for older people with care needs in Europe. Our study focuses on investigating how gender, widowhood and living arrangement associate with the probability of receiving home and community-based care, while accounting for the shorter-term associations of transitions into widowhood (bereavement) and living alone, as well as the longer–term associations of being widowed and living alone. We use comparative, longitudinal data from the Survey of Health, Ageing and Retirement in Europe (collected between 2004 and 2015 in 15 countries) specifying sex-disaggregated random-effects within-between models, which allow us to examine both cross-sectional and longitudinal associations among widowhood, living arrangements and community-based care use. We find widowhood and living alone are independently associated with care use for both older women and men, while bereavement is associated with higher probability of care use only for women. Socio-economic status was associated with care use for older women, but not for men in our sample. The gender-specific associations we identify have important implications for fairness in European long-term care systems. They can inform improved care targeting towards individuals with limited informal care resources (e.g. bereaved older men) and lower socio-economic status, who are particularly vulnerable to experiencing unmet care needs. Gender differences are attenuated in countries that support formal care provision, suggesting gender equity can be promoted by decoupling access to care from household and family circumstances.

**Supplementary Information:**

The online version contains supplementary material available at 10.1007/s10433-022-00717-y.

## Background and objectives

Increasing numbers of older adults spend longer periods with chronic illness and functional limitations, rendering them reliant on formal and informal support to continue living independently in the community (Spasova et al. 2018). Large differences in the availability and affordability of home and community-based care services—commonly referred to as ‘long-term care’ in the European context and throughout this paper—lead to marked variability in how older people in need of care can access such support across European countries (Oliveira and Llena-Nozal 2020; Rodrigues et al. 2018). Such inequalities have been revealed and likely deepened by the Covid-19 pandemic and some of the control measures imposed in its aftermath. Furthermore, taken together, key life events and socio-economic dynamics create variability in care needs and care use patterns between different groups within those same countries, such that available care is not always used by those in most need, but rather by those most able to afford and access it (Floridi et al. 2020; Ilinca et al. 2017).

The experience of ageing, as well as functional and socio-economic trajectories, differ markedly between women and men (Phillips et al. 2016). Due to higher longevity, women are significantly more likely to outlive their male spouses, leading to a sex imbalance in widowhood and living arrangements in old age. In addition, the experience of widowhood affects men and women differently, both with respect to care needs and in relation to accessibility of care resources (Arber et al. 2003). Women live longer than men, but more of those added years are lived with disability and functional impairment (Leveille et al. 2000). They are therefore more reliant on care resources in later life but often less able to afford care. Gender differences in factors such as income, wealth and social capital, while relevant across age groups, are particularly pronounced for current old age cohorts: in Europe, the share of individuals at risk of poverty is highest among women aged 75 years or older, partially reflecting the higher share of older women who are widowed (Eurostat 2019a, b).

We focus on three important dimensions of unequal care use in later life: sex/gender, widowhood and living arrangements. Gender is intertwined with every aspect of the experience of ageing and often inseparable from patterns of socio-economic inequality in old age (Van der Linden et al. 2019). In this study, we aim to understand how widowhood and living arrangements are associated with the probability of receiving home and community-based formal and informal care for older people with care needs and how sex/gender and socio-economic differences affect this association.

This analysis is rendered both timely and highly relevant by changing patterns of co-habitation among older age cohorts (i.e. decrease in intergenerational households) and the increasing necessity to ensure all older adults are facilitated to live independently, irrespective of their marital and partnership status. In addition, it is important to understand whether transitions into widowhood and changes in living arrangements in old age are independently associated with care use, and if such patterns are gendered, in order to correctly target support services and provide guidance to families and other informal caregivers.

Throughout our analysis, we place differences between women and men at the core of our analytical strategy, recognizing the intersectional nature of different sources of disadvantage in old age (Phillips et al. 2020). In recognition of the high variability of care supply and demand patterns across Europe, we also present disaggregated results for four country clusters, which share key commonalities in the organization and structure of their long-term care systems.

### Widowhood, living arrangements and care needs in later life

Widowhood is understood as the long-term and ongoing state of having lost a spouse through death, and the new social status that results from this transition. It is distinguished from bereavement (transition into widowhood) which refers to the experience of the death of a spouse and the status of mourning that follows it, generally with shorter-term consequences (Bennett and Soulsby 2012). Across European countries, widowhood is overwhelmingly experienced by women and increases considerably with age. An average of 3.5% of men aged 60 to 64 in Europe are widowers (ranging from 2% in Sweden to 6% in Romania), while 14% of women in the same age groups are widows (with considerable country variation, between 6% in Sweden and 24% in Bulgaria). After the age of 85, approximately 40% of older men and three in every four older women are widowed in Europe (Eurostat Database 2019).

Widowhood and bereavement are among the most distressing life events individuals can experience, with profound consequences on functioning and care needs for older people. Both widows (women who have lost a spouse) and widowers (men who have lost a spouse) often experience lower psychosocial wellbeing, physical and mental health, changes in their personal relationships and social interactions, as well as negative economic consequences and financial strain (Soulsby and Bennett 2017; Bíró 2013). However, gender is an important determinant for the experience of and adjustment to late life widowhood (Arber et al. 2003; King et al. 2019; Kung 2020). Whereas women are more vulnerable to financial distress and poverty after the loss of a spouse (Gillen et al. 2009; Bíró 2013; Streeter 2020), the adverse mental and physical health outcomes associated with widowhood are more pronounced for men who are more likely to be depressed and experience subsequent higher mortality (Lee et al. 2001; Bennett et al. 2005; Streeter 2020). In addition, widowhood triggers changes in living arrangements. As the majority of older people in Europe live solely with a spouse or partner (Eurostat 2019a, b), widowhood is often equivalent to a transition to living alone in old age. This exposes those widowed to increased risk of loneliness and social isolation and subsequent declines in functioning, physical and mental health at a time when emotional and psychological support are essential for maintaining morale and coping with grief (de Jong Gierveld et al. 2012; Nakagomi et al. 2020). Living alone is also considerably more common among older women in Europe. On average, 40% of women aged 65 and above in Europe, as compared to 20% of men of the same age lived alone in 2017. The share of older women living alone surpasses 50% in Denmark and Estonia and is lower than 30% in Luxembourg or Spain (Eurostat 2019a, b).

### Widowhood, living arrangements and care resources in later life

The loss of a spouse has a double impact: it may increase older adults’ need for care and support and can also represent the loss of a key caregiver. A significant share of informal caregiving in Europe today is provided within the household, most often by a spouse (Bertogg and Strauss 2020), and while large gender gaps in informal caregiving are apparent in adulthood, the shares of older men that engage in caregiving increases with age to equal and even surpass that of women (WHO, 2018). According to data for the EU-27, approximately 5% of men aged 65 to 74 and 6% of men aged 75 and over provide care inside the household, with the corresponding shares for women of 5% in both age groups (Eurostat 2020). However, while men are more likely to be cared for by their spouse or informal caregivers only, women are more likely to rely on formal care services and receive care from multiple formal and informal caregivers, even when cared for by their spouse (Bertogg and Strauss 2020; Ilinca et al 2017; Schmidt 2017). After the death of a spouse, surviving partners are faced with needing to manage household tasks that were shared when living as a couple and all personal care tasks. As a result, for older women and men who experience functional limitations, dependence on home and community-based support (both formal and informal) often increases with widowhood and when living alone (Pimouguet et al. 2016). Those who cannot rely on well-developed social support networks or are unable to afford needed care, are particularly vulnerable to experiencing unmet care needs and see their ability to continue living in the community severely affected (Thomeer et al. 2016), especially shortly after the loss of a spouse (Nihtila and Martikaainen 2008).

### Gender, income and educational inequalities in care use

Lower income older individuals are more likely to report care needs in later life, while at the same time they are less able to afford needed care (Oliveira Hashiguchi and Llena-Nozal 2020; European Commission and Social Protection Committee, 2021). As a result, they more often rely on informally provided care and report unmet care needs linked with an inability to pay, although such inequalities are more contained in de-familized care systems (Eurofound, 2020; Floridi et al. 2020; Ilinca et al. 2017). Education achievement is closely entwined with income and socio-economic status and can additionally affect care use through increasing individual ability to navigate complex care systems and pathways. Following an accumulation of disadvantage across the life course, older women are considerably more vulnerable, as they are more likely both to have primary education only, low incomes and face poverty in old age (particularly in the event of widowhood or family dissolution) and to require care in later life (Bíró 2013; Streeter 2020; European Institute for Gender Equality 2016).

### Study objectives

In combination, widowhood and living alone expose older people to a series of health, emotional, social and practical challenges that increase their need for support while concomitantly depriving them of key caring resources. However, to the best of our knowledge, no study has attempted to disentangle how widowhood and living arrangement transitions are associated with the probability of receiving needed home and community-based care. Because they co-occur so frequently and are so closely related to all the established determinants of care use (health and functionality, economic resources and social ties), separating their influence can be a complex exercise.

The aim of our study is therefore, to explore the complex pattern of associations between widowhood, living arrangements and use of home and community-based care for older men and women. We state three inter-related objectives. Firstly, to examine if widowhood and living alone are independent predictors of the probability of using care in the community for older women and men with care needs. Secondly, we investigate gender specific patterns in the association of marital status and living arrangements with care use patterns, with particular attention to the associations of transitions into widowhood and living alone. Finally, we aim to account for the influence of financial and human capital (i.e. educational achievement), and reflect on how disadvantage in these dimensions can overlap with sex to influence patterns of home and community-based care use for older women and men with care needs.

To this end, we employ a random-effects within-between model specification which allows us to estimate both cross-sectional and longitudinal associations, including for time invariant variables that are of interest to our study, without relying on strong exogeneity assumptions. This approach overcomes the shortcomings of more common fixed- and random-effects specifications and is gaining increasing attention in political science, health research and economics studies (Rummo et al. 2020; Fairbrother et al. 2019; Schumann 2020).

## Research design and methods

### Analysis sample

We use data from the Survey of Health, Ageing and Retirement in Europe (SHARE) a multidisciplinary and cross-national database including information on health, socio-economic status and social and family networks of older Europeans (Börsch-Supan et al. 2013). We maintain for the analysis only data from the panel waves of the survey, collected in 2004–5 (wave 1), 2006–7 (wave 2), 2011 (wave 4), 2013 (wave 5) and 2015 (wave 6). Waves 3 and 7 (collected in 2009 and 2017), which mainly include retrospective data and life histories, were excluded from the present analysis. While wave 7 includes regular panel data which could have supplemented our sample, the complete lack of data in some countries and the very small samples in others precluded its inclusion in the final analysis sample.

We also excluded all observations from countries that have not participated in at least two consecutive panel waves, leading to coverage of 15 European countries, which were clustered in four different long-term care regimes following a typology previously used in the literature (Albertini and Pavolini 2017; Carrieri et al. 2017). While numerous long-term care system typologies exist, each emphasizing different system characteristics (Ariaans et al 2021; Damiani et al 2011; Kraus et al 2010), the typology used in the present analysis focuses primarily on three dimensions that are essential for the availability and accessibility of different community-based care types: the extent of public financing for LTC; the degree of familialization of care provision and the balance between formal and informal care provision; and the orientation towards in-kind versus cash benefits (a detailed description of the main characteristics defining each cluster is presented as supplementary material—Online resource 1). According to this categorization,  we obtain four country clusters a) Continental (Austria, Germany, France, Belgium and Switzerland); b) Nordic (Sweden, Denmark, Netherlands); c) Southern (Italy, Spain, Greece) and d) Eastern (Czech Republic, Slovenia, Poland, Estonia).

We further restricted the sample to those individuals who are aged 60 years or older at least at one point in the panel and who report continued care needs for at least two consecutive panel waves. Care needs are assessed as the presence of one or more limitations in activities of daily living (ADL) and independent activities of daily living (IADL), three or more mobility, arm function and fine motor limitations or diagnosed cognitive impairment (Alzheimer’s or dementia diagnosed by a physician). By focusing on those older individuals (population of interest) who experience sustained functional limitations assessed through several indicators, we are able to investigate how changes in marital status and living arrangement associate with care use, while limiting the possibility that these are confounded by changes in care needs status. Moreover, in order to ensure we can separate the influence of widowhood from that of any marital status transition, we excluded from the sample those individuals who reported living in a registered partnership, never being married or being divorced.

Our final analytic sample includes 32,139 observations from 12,733 individuals, describing an unbalanced panel (see Supplementary material—Online resource 2). The sample includes 21,972 observations (representing 68.4% of the total sample) from 8561 women and 10,167 observations (representing 31.6%) from 4174 men.

### Dependent variable

Our dependent variable is a binary indicator of whether an individual receives any type of care in their own home, including informal care from family members, neighbors and members of one’s social network or formal care, provided by care professionals. This includes support with personal care as well as support for domestic tasks, household maintenance nutrition and social participation (European Commission and Social Protection Committee, 2021; World Health Organization, 2021). The variable captures care provided by persons residing either within the same household as the care recipient or outside the household and takes a value of 1 if an individual responded ‘Yes’ to at least one of the following survey items:

- Thinking about the last twelve months, has any family member from outside the household, any friend or neighbor given you/your partner any kind of help [*with personal care or domestic tasks*]?—only asked of the family respondent. To avoid overestimating the amount of care received by older individuals living in couples, we have not assigned positive value to co-residing partners of family respondents.
- Is there someone living in this household who has helped you regularly during the last twelve months with personal care, such as washing, getting out of bed, or dressing?
- During the last twelve months, did you receive in your own home any professional or paid [*care*] services due to a physical, mental, emotional or memory problem? [*including personal care, domestic tasks, meals-on-wheels*]—included in waves 1, 2, 5 and 6.

The choice of an outcome variable encompassing formal and informal care reflects our interest in exploring and informing academic and policy discussion on the care resources available for older people in the community more generally, rather than the characteristics of any particular care type. This broader conceptualization aligns with the increasingly common occurrence of mixed care arrangements in Europe and is particularly relevant for understanding what care resources are available to meet care needs of people who are widowed and living alone, and therefore have no access to care provided inside the household or by a spouse or partner.

### Independent variables

The main exposures of interest for our study are sex, widowhood and living arrangements. A binary variable that identifies widowhood was generated based on self-reported marital status in each panel wave—married living with spouse, married not living with spouse or widowed. Living arrangements are described in our analysis by two separate variables: a binary variable for living alone and a continuous variable for household size (i.e. number of household members, irrespective of their familial relationships with the respondent).

Socio-economic capital is captured by two predictors: income quartile and education achievement. We calculated country-specific quartiles for equivalized net household income, obtained through the aggregation of all household level income components (including social benefits). Level of education is a binary indicator for individuals whose highest level of completed education is primary education only or no formal education (derived from ISCED codes, harmonized across countries) as opposed to having secondary or tertiary education.

We further controlled for age and a set of physical and mental health status indicators, which include: poor or very poor self-reported health; the self-reported number of chronic conditions as diagnosed by a physician, and poor mental health (defined as a EURO-D score higher than 3). While our sample has already been selected to include only individuals with care needs, we further included covariates for physical functioning, i.e., the number of limitations with ADL and IADL, which allowed us to account for the influence of severe care needs.

### Analytical approach

Data are hierarchically structured, with each individual observed on several occasions over time. This structure is significant methodologically and substantively, as we are interested in modelling both the association of widowhood and living arrangements with the probability to receive care across the population and the association between transitioning into widowhood and care use for individuals in the population. In order to examine both cross-sectional (between individuals) and longitudinal (within individuals, over time) associations, rather than assuming they are equivalent, we employ the random effects within-between model—REWB (Allison 2009; Bell et al. 2019; Schunk and Perales 2017). REWB is a multi-level modelling approach, which is gaining increasing attention in social and political science due to its ability to combine the strengths of more established fixed- and random-effects estimation approaches. Fixed-effects models are commonly considered the gold standard for longitudinal data analysis as they provide consistent estimates of within-cluster effects even in the presence of unobserved heterogeneity. However, they are limited in that they cannot estimate effects of variables that do not vary within clusters. Conversely, random effects specifications can be used to identify the effect of cluster-invariant variables but only under strict exogeneity assumptions. Similar to random-effects models, the REWB models allow for the inclusion of cluster-invariant variables, which are of significant interest in our study. This is the case for level of educational which is virtually constant for older individuals with care needs, considered in our study.

Throughout, we run sex disaggregated models, presenting results for women and men independently. This approach allows us to evaluate whether widowhood and living alone are dissimilarly associated with care receiving for women and for men and to reflect on the difference in association of socio-economic disadvantage between sexes.

## Results

Descriptive statistics for the study sample, disaggregated by sex, are presented in Table 1. Our sample restriction to older people with care needs only and the higher prevalence of functional limitations and care needs among older women explain the large imbalance in sex distribution.

**Table 1:** Descriptive statistics for study sample, by sex

	Women		Men		Min	Max
	% (Mean)	*N*	% (Mean)	*N*		
*Receives care*					0	1
No	48.17	10,585	52.65	5353		
Yes	51.83	11,387	47.36	4814		
*Age*	(74.98)	21,972	(75.02)	10,167	49	106
Poor self-reported health	75.87	16,671	78.34	7965	0	1
No. of chronic conditions	(2.96)	21,972	(2.88)	10,167	0	14
No. ADL limitations	(0.91)	21,972	(1.1)	10,167	0	6
No. IADL limitations	(1.64)	21,972	(1.70)	10,167	0	9
Poor mental health	58.32	12,815	48.83	4965		
*Widowed*	44.24	9720	14.84	1509	0	1
Transitions into widowhood	7.3	567	3.24	168		
Transitions out of widowhood	0.27	15	0.25	2		
Household size	(1.88)	21,972	(2.15)	10,167	1	11
Lives alone	35.87	7881	13.89	1412		
Primary or no education^a^	45.80	10,063	36.79	3740	0	1
*Income quartile*^a^					1	4
First	25.95	5701	18.31	1862		
Second	25.85	5680	22.61	2299		
Third	24.50	5383	28.37	2884		
Forth	23.70	5208	30.71	3122		
*Welfare cluster*^b^						
Continental	34.38	7554	36.83	3744		
Nordic	11.62	2553	12.39	1260		
Southern	26.73	5874	24.19	2459		
Eastern	27.27	5991	26.60	2704		

Unweighted pooled data (SHARE 2004–2015)

^a^Income quartiles are calculated at the country level for the sample aged 60 and above

^b^Welfare clusters include the following countries: Continental—AT, DE, FR, BE, CH,; Nordic—SW, DK, NL, Southern—IT, ES, EL, Eastern—CZ, PL, SLO, EST

Despite this restriction, the sex/gender patterns we observe closely reflect results from previous population-based studies. Only half of the older people in our sample receive care although all are in need of care according to the measured indicators of functionality, indicating a considerable proportion of unmet care needs in older European populations. Women are on average more likely to receive care, although average age and the distribution of care needs are comparable across sexes. The share of women who transition into widowhood and who live alone is substantially higher than that of men, who live in households of larger average size. Differences between sexes are also apparent in socio-economic conditions. Fewer men report only primary or no education. While women are relatively evenly distributed across income quartiles, older men in our sample are concentrated in richer income quartiles.

Table 2 summarizes the results of our analysis. We find widowhood is significantly and positively associated with the probability of receiving long-term care for both women and men (between effect), while the transition into widowhood increases the likelihood of receiving care only for older women (within effect). In other words, while both widows and widowers have a higher probability of receiving care than older individuals with partners, bereavement (i.e. the transition from marriage to widowhood) triggers an average increase in care use only for older women (Model 1, unadjusted). Because widowhood is associated with changes in care needs and in financial and human capital, albeit differently across sexes, we further account for this associations. Our results are robust, controlling for potential confounding from a complex set of health and functional status indicators, including severity of care needs (Model 2) and of income and level of education (Model 3). The direction and statistical significance of this association is confirmed when also considering living arrangements (Model 4), although the strength of the association is markedly reduced. Living alone is a significant predictor of the probability of receiving care for both women and men, with a stronger relationship with care use than observed for widowhood or for bereavement. Despite a relatively high correlation between the widowhood and living alone indicators, our results are robust and remain stable in a model including only these two covariates (results available on request). Household size is only weakly associated with care use for women, and not at all for men (between effects). We find no evidence for an association between changes in household size or transitioning to living alone and the care use by older women and men.

**Table 2:** Results from nested random between-within effects models on probability to receive care, by sex (odds ratios)

	(1)		(2)		(3)		(4)	
	Women	Men	Women	Men	Women	Men	Women	Men
Widowed, within effect	1.632***	1.283	1.465**	1.072	1.486**	1.086	1.699***	0.976
Widowed, between effect	2.267***	2.323***	2.095***	2.343***	2.143***	2.375***	1.388***	1.550**
Poor health, WE			1.154*	1.316**	1.155*	1.317**	1.156*	1.322**
Poor health, BE			1.629***	1.497***	1.660***	1.512***	1.667***	1.502***
Mental health, WE			1.327***	1.348***	1.328***	1.350***	1.330***	1.345***
Mental health, BE			1.121	1.158	1.135*	1.163	1.133*	1.147
Chronic conditions, WE			1.125***	1.043	1.123***	1.043	1.123***	1.043
Chronic conditions, BE			1.148***	1.062**	1.147***	1.061**	1.139***	1.063**
ADL limitations, WE			1.209***	1.322***	1.210***	1.323***	1.208***	1.322***
ADL limitations, BE			1.274***	1.561***	1.271***	1.557***	1.268***	1.554***
IADL limitations, WE			1.234***	1.235***	1.235***	1.235***	1.233***	1.233***
IADL limitations, BE			1.408***	1.351***	1.415***	1.356***	1.437***	1.359***
Primary or no education					0.859**	0.941	0.873**	0.952
Income, WE					1.047*	1.054	1.046*	1.055
Income, BE					1.052*	1.065	1.056*	1.055
Live alone, WE							0.833	1.300
Live alone, BE							1.727***	1.670**
Household size, WE							1.015	1.118
Household size, BE							0.921*	0.937
No. of observations	21,972	10,167	21,972	10,167	21,972	10,167	21,972	10,167
No. of individuals	8561	4172	8561	4172	8561	4172	8561	4172

Exponentiated coefficients. Unweighted results. All models include age and country controls

^*^p < 0.05; **p < 0.01; ***p < 0.001

Our results further confirm previous findings of a differential influence of socio-economic status indicators across sexes. The probability of receiving care is significantly associated with income and level of education only for older women in our sample, while no significant associations are identified for older men.

We next turn our attention to the possibility that the above results are moderated by country specific characteristics and institutional factors that are not fully captured by country specific dummy variables. In Table 3, we present the results of separate analyses of country clusters organized along the care regime typology. For Continental and Southern European care regimes, the results are very similar to those reported for the pooled European sample. Widowhood is a positive and significant predictor of care use for both women and men, while transitions into widowhood (within effect) only affect care use by older women. In Eastern European countries (Czech Republic, Slovenia, Poland, Estonia), we find evidence of an association of widowhood and bereavement only for older women, while in Nordic countries (Sweden, Denmark, Netherlands) the association is not statistically significant for either gender. Living alone is associated with higher probability of receiving care for women across all care regimes, with somewhat stronger association than widowhood, while results for men vary between country clusters. Finally, we find a highly variable pattern of association between socio-economic status indicators and care use across sexes and care regimes.

**Table 3:** Results from random between-within effects models on probability to receive care, by sex and welfare regime (odds ratios)

	Continental		Nordic		Southern		Eastern	
	Women	Men	Women	Men	Women	Men	Women	Men
Widowed, within effect	2.021*	0.898	0.776	0.722	1.663*	1.548	1.836*	0.534
Widowed, between effect	1.565***	1.631*	1.002	1.520	1.384*	2.070*	1.299*	1.208
Live alone, WE	1.212	1.694	1.721	1.873	0.534*	1.008	0.741	1.725
Live alone, BE	1.718**	1.502	4.271***	2.942	1.673**	0.784	1.536**	2.510**
Primary or no education	0.865	0.967	1.013	1.055	0.776*	0.902	0.910	0.836
Income, WE	1.045	1.068	1.081	0.900	1.069*	1.111*	0.982	0.925
Income, BE	1.164**	1.159	1.297*	1.084	1.025	1.027	0.979	0.997
No. of observations	7554	3744	2553	1260	5874	2459	5991	2704
No. of individuals	2858	1489	1014	533	2251	1014	2438	1136

Exponentiated coefficients. Unweighted results. All models include age, country dummies, poor self-reported health, Poor mental health, number of chronic conditions, ADL limitations, IADL limitations, household size (results not reported)

^*^p < 0.05; **p < 0.01; ***p < 0.001

## Discussion and implications

Our study set out to explore how marital status and living arrangements in later life are associated with home and community-based care use among older Europeans with care needs, while paying particular attention to the role of transitions into widowhood and living alone, and the role of gender. Because widowhood and living alone co-occur frequently in current older age cohorts, it has proved difficult to disentangle their influence on care use. Our results show that being widowed and living alone have overlapping but independent associations with long-term care use and are both associated with increases in the probability of using care in later life for women and men alike (Objective 1). While we find living alone has a stronger relationship than widowhood with the probability of care use for both women and men, the two are comparable. Importantly, our results suggest the cumulative impact of being widowed and living alone places certain groups of older individuals (e.g. bereaved men) in positions of increased vulnerability and exposes them to being at higher risk of unmet care needs. As prevailing trends in marriage and living arrangements will lead to an increasing number of widows and widowers facing the possibility of old age dependency without being able to rely on support from members of their household, long-term care systems must respond by increasing service availability and targeting older individuals with limited informal care resources. Older women in need of care are more likely to use a combination of informal and professional care while older men more often rely on informal care only (Carvalho et al. 2019), which could better insulate the former from unmet needs arising from the death of their spouse. Many European countries already implement carer-sensitive policies (prioritizing access for individuals without informal support within the household). Our results, however, point to the need to also facilitate access to professional home care for those who have been widowed, irrespective of their living arrangements. This could be achieved by proactively offering a needs assessment immediately after the transition into widowhood, similarly to what some systems already provide to informal carers.

The second objective of our study was to investigate gender specific associations of marital status and living arrangements with care use patterns, by separating the shorter-term influence of transitions into widowhood (bereavement) and living alone from the long-term influence of the state of being widowed and living alone. We found bereavement is significantly associated with increased care use only for older women, after controlling for the intensity care needs. This suggests older widows are better able to access care resources following bereavement, whereas older widowers are less likely to do so. The literature suggests a number of potential causal mechanisms. Firstly, widowers have on average less extensive social networks and less contact with children following bereavement (Soulsby and Bennett 2015) which may limit the availability of informal care resources. Secondly, older men are less likely to seek and participate in community-based care programs (Milligan et al. 2013). Finally, the surviving spouses in our study (i.e. the widows and widowers) were likely previously providing informal care to their deceased spouse. There is evidence that households where the wife is the informal carer tend to receive less formal care service due to persistent gender stereotypes of women’s roles as caregivers and less confidence on the ability of male spouses to provide care (Carvalho et al. 2019; Schmidt 2017; Larsson et al. 2014). Further research should attempt to establish whether frail older women regularly forgo needed care if their spouse has higher support needs and how long-term care services are allocated within households where both spouses are in need of support, albeit at different intensity levels.

Our third objective was to reflect on the intersection of sex and access to financial and social capital in influencing patterns of home and community-based care use for older women and men with care needs. While the extant literature establishes an association between lower income and lower formal care use (Floridi et al. 2020; Crepaldi 2009; Ilinca et al, 2017), we found socio-economic status indicators were associated with care use only for older women, suggesting a modifying effect of sex and a disproportionate association of lower income on access to care for older women. Our results further point to having no education or only primary education as a barrier in accessing care that goes beyond the issue of affordability (i.e. after controlling for household income and changes in income). Such findings confirm previous results (Crepaldi et al 2009) and raise concerns with respect to the ability of older people, particularly older women in these cohorts who have lower rates of completed secondary and tertiary education, to navigate complex procedures for identifying and accessing formal care services to which they are entitled.

We note four limitations of our study. First, the results presented rely on data aggregation at European or regional country clustering levels, which obscures important national level differences in care systems, population and care use characteristics, as well as in sampling and data quality. We were limited in our ability to carry out country-specific analyses by the size of the longitudinal sample in SHARE. To fill in the resulting gap in detail we encourage the replication of our approach using richer, national level datasets, where they are available. The second limitation arises from the inability to control for the intensity of care received and for the potential effect of time-varying health and family characteristics on care use. Consequently, we cannot establish whether widows and widowers who receive care do so at a level commensurate with their need for support. It is likely therefore, that our results underestimate the number of individuals who forgo needed care and the prevalence of care poverty (inadequate coverage of care needs) in certain population groups. Thirdly, we have relied in the present analysis on a composite measure of community-based care use which merges into one indicator variable both formal and informal care received by respondents. While these forms of care are complementary, it is likely associations with several variables of interest differ between formal and informal care and across country clusters, with variations in availability of different care types. It is therefore important to interpret the results with a general perspective of access to community-based support for older individuals with care needs and to focus future research efforts on identifying specificities and differences in patterns of use for specific types of support. Finally, the comprehensive care use indicator we have employed could not be constructed uniformly across all SHARE waves, as the question on home-based formal care use has not been included in the wave 4 instrument. This limits comparability of the indicator across waves but not across individuals within waves or across country clusters. As our results are generally robust to the exclusion of all wave 4 data from the analysis, we have opted to maintain the widest possible sample of individuals as this allows us coverage of less studied Eastern European country samples.

It is noteworthy that our results point to marked differences across clusters of countries, grouped according to core characteristics of national long-term care systems. More specifically, we find gender differences in the association of widowhood and living arrangements on probability to receive long-term care are attenuated in countries that emphasize *defamilialization (of care) through public provision*, shifting responsibility for care from the family towards the state (Saraceno 2016). In countries belonging to the Nordic cluster, the State recognizes and assumes responsibility for fulfilling individual care of older adults with functional impairment, therefore decoupling, to a large extent, access to care from household and family circumstances. The association between socio-economic status indicators and care use varies across country clusters. While partly attributable to large differences in cultural and social underpinnings across country clusters, such variability also highlights remaining gaps in equity achievement across European care systems.

Our study is concerned with identifying differences between groups and inequalities in accessing care, but we believe important implications for fairness in long-term care systems (i.e. inequities in care use) can be derived from our findings. An important step has been made in the European context towards recognizing that ‘everyone has a right to affordable long-term care services of good quality, in particular home-care and community-based services’ (Principle 18) with the adoption of the European Pillar of Social Rights (2017). At the same time, the Covid-19 pandemic has revealed important vulnerabilities and large gaps in coverage, accessibility and quality of care provision across Europe. As equity achievements are increasingly recognized as a key priority for national long-term care systems in Europe, and in view of the elaboration of a European Care Strategy, a better understanding of gender and socio-economic differences in care use will play a crucial role in defining a pathway towards ensuring care is accessible to all those who need it (European Commission and Social Protection Committee 2021). This is particularly relevant in view of the increased reliance on carer-sensitive policies in European countries and a tendency to tighten eligibility criteria for accessing publicly financed care (limiting access for individuals with low and moderate care needs), as part of a concerted effort to ensure fiscal sustainability of long-term care systems in the face of population ageing (EIGE 2020). It is also noteworthy that some European countries restrict access to publicly funded service, especially when funded through social insurance schemes, to individuals who have not been employed or in precarious employment. As these are more likely to be women, coverage of community-based formal care services may be more limited for them. Similarly, capping of publicly funded in-kind home care services to a number of hours that does not sufficiently cover the needs of individuals with moderate and severe care needs, leads to increased financial burden on care recipient who must purchase additional services and added pressure on family caregivers to cover care gaps (EIGE 2020; Spasova et al 2018). Taken together, such policies place older women consistently at a disadvantage, especially when compounded with other potential sources of disadvantage like widowhood and living alone. The equity impact of carer-sensitive policies on care use has not been extensively studied (Bakx et al. 2015; Ilinca et al. 2017), and further analyses are needed, but it is clear the burden of increased informal caregiving that is related to carer-sensitive policies is primarily born by women (EIGE 2020). Our results indirectly point to a potential downstream effect of eligibility criteria and service targeting practices for formal care to older couples, before bereavement and widowhood. Even when eligibility is not explicitly conditioned on household characteristics, the allocation of scarce resources may still be underpinned by stereotyping the spouses’ ability to provide care. Because older women may be perceived as more natural and able caregivers they are less likely to receive additional support, while men providing care are more likely to share care tasks with other formal and informal caregivers (Bertogg and Strauss 2020; Schmidt 2017). The findings presented here suggest that care use patterns following bereavement and widowhood should be understood in light of these pervasive gender inequalities and deeply embedded gender roles with respect to care and caregiving. Assessments of care needs should explicitly account for the increased vulnerability of individuals who experience such significant life transitions and concomitantly placed at the intersection of various other sources of disadvantage.

A better understanding of how community-based caring resources are accessed by older women and men is essential to inform policies that address care gaps for groups at risk of experiencing unmet care needs and to ensure all older people are able to continue living independently and with dignity in their communities. Our study highlights, once more, that at the core of this understanding must lie an appreciation of the role of gender in the experience of ageing, of key life-course transitions and of household and family characteristics.

## Supplementary Information

Below is the link to the electronic supplementary material.

Supplementary file1 (DOCX 15 kb)
