# Supplementary material for: Gender differences in access to community-based care: a longitudinal analysis of widowhood and living arrangements
Source: Eur J Ageing. 2022 Jul 27;19(4):1339–50. doi: 10.1007/s10433-022-00717-y (PMC9326144; doi:10.1007/s10433-022-00717-y)
Supplement: Supplementary file 1 — Supplementary file1 (DOCX 15 kb) [file 10433_2022_717_MOESM1_ESM.docx]

**Supplementary material**

Online Resource 1 - Typology of European welfare regimes

| **Welfare regimes** | **Countries** | **Characteristics** |
| --- | --- | --- |
| **Nordic** | Sweden, Denmark, Netherlands | High public expenditure on LTC; low intensity but widespread access to informal caregiving; limited cash-for-care benefits and high provision of care services. |
| **Continental** | Austria, Germany, France, Belgium, Switzerland | Medium public expenditure on LTC; medium intensity of informal caregiving; cash-for-care benefits and medium provision of care services |
| **Southern** | Italy, Spain, Greece | Medium to low public financing on LTC; high intensity informal caregiving; cash-for-care benefits and low provision of care services |
| **Eastern** | Czech Republic, Slovenia, Poland, Estonia | Medium to low public financing on LTC; high intensity informal caregiving; low provision of cash-for-care and care services |

Source: Adapted from Carrieri, Di Novi & Orso (2017) and Albertini & Pavolini (2017).

Note: The classification of the Netherlands along with Scandianavian countries in the Nordic cluster, while departing from the classical composition of the Scandinavian cluster, reflects strong commonalities in high public investment in LTC and the emphasis on in-kind benefits (direct provision of care services) as opposed to cash benefits. A classification of the Netherlands along with other Nordic countries is also proposed by Damiani et al 2011 and Kraus et al 2010

**Supplementary material**

Online Resource 2. Panel structure of the analysis sample, by gender

|  | **Men** | | |  | **Women** | | |
| --- | --- | --- | --- | --- | --- | --- | --- |
| Participation in | N. individuals | N. observations | *% Male sample* |  | N. individuals | N. observations | *% Female sample* |
| 2 waves | 2779 | 5558 | *54.7* |  | 5107 | 10214 | *46.5* |
| 3 waves | 1065 | 3195 | *31.4* |  | 2460 | 7380 | *33.6* |
| 4 waves | 226 | 904 | *8.9* |  | 592 | 2368 | *10.8* |
| 5 waves | 102 | 510 | *5.0* |  | 402 | 2010 | *9.1* |
| **Total** | **4172** | **10167** | ***100*** |  | **8561** | **21972** | ***100*** |
